# Supplementary material for: Indole-3-acetaldehyde dehydrogenase-dependent auxin synthesis contributes to virulence of Pseudomonas syringae strain DC3000
Source: PLoS Pathog. 2018 Jan 2;14(1):e1006811. doi: 10.1371/journal.ppat.1006811 (PMC5766252; doi:10.1371/journal.ppat.1006811)
Supplement: S1 Text — (DOCX) [file ppat.1006811.s001.docx]

**S1 Text**

**Materials and Methods for IAA Extraction and Quantification**

**Materials**

Indole-3-acetic acid and indole-2,4,5,6,7-d_5_-3-acetic acid (d_5_-IAA, internal standard) were purchased commercially from Sigma-Aldrich (St. Louis, MO; Cat No. I3750-25G-A) and CDN Isotopes (Pointe-Claire, Quebec; Cat No. D-2203), respectively. Methanol and acetonitrile (HPLC-grade) were sourced from J.T. Baker (Avantor Performance Materials) and LC-MS grade water was purchased from Honeywell Research Chemicals. Individual stock solutions of unlabeled and labeled compound were prepared in 50% methanol and stored at -80 °C. Standard solutions were prepared fresh in 30% methanol in the linear concentration ranges of 32 pM to 10 µM. An internal standard solution was prepared in 30% methanol containing 2.5 µM d_5_-IAA.

**LC-MS/MS Instrumentation**

Clarified samples were analyzed on a Eksigent ekspert™ microLC 200 coupled to a Sciex 6500 QTrap® (Framingham, MA) operated with positive-ion electrospray ionization. The LC separation was achieved using a Waters (Milford, MA) Acquity UPLC® BEH C18 1.0 × 100 mm, 1.7 µm column kept at 50 °C with a flow rate of 15 µL/min while the autosampler was set at 8 °C. The mobile phases were 0.1% acetic acid and 3:1 acetonitrile:methanol containing 0.1% acetic acid running a gradient of 20% B for 4 minutes ramping to 70% B at 7 minutes, increasing to 95% B at 7.5 minutes, holding for 5.5 minutes, then re-equilibrate at initial conditions at 13.5 minutes for 10 minutes (total runtime is 23.5 minutes). Data analysis was completed using MultiQuant 3.0.2 (AB Sciex) and normalizing the unlabeled IAA peak area to the labeled d_5_-IAA peak area. Quantitation of IAA was accomplished by observing the multiple reaction monitoring (MRM) transition of 176.0 → 130.0 *m/z* relative to the 181.0 → 134.0 *m/z* transition for d_5_-IAA. Calibration curves were linear (*r* values = >0.99) within the ranges provided above applying a 1/x weighting scheme.

**IAA Extraction from Bacterial Cultures**

Frozen bacterial cultures were thawed on ice. A 10 µL aliquot of thawed culture was transferred to a microcentrifuge tube containing 10 µL of 2.5 µM d_5_-IAA and 180 µL 30% methanol Samples were vortexed for 30 s, then filtered through 0.8 µm PES spin-filters (Sartorious). 40 µL of clarified supernatant was transferred to HPLC vials; injected 2 µL of sample onto column.

**Compound-dependent parameters**

| Compound | Retention time (min) | DP (V) | EP (V) | CE (V) | CXP (V) |
| --- | --- | --- | --- | --- | --- |
| IAA | 9.7 | 23 | 7 | 40 | 19 |
| d_5_-IAA | 9.7 | 23 | 7 | 25 | 19 |

*Source settings*

Ionspray voltage: 4500 V

Curtain gas: 15

Gas 1: 35

Gas 2: 35
