# Supplementary material for: Indole-3-acetaldehyde dehydrogenase-dependent auxin synthesis contributes to virulence of Pseudomonas syringae strain DC3000
Source: PLoS Pathog. 2018 Jan 2;14(1):e1006811. doi: 10.1371/journal.ppat.1006811 (PMC5766252; doi:10.1371/journal.ppat.1006811)
Supplement: S1 Table — (DOCX) [file ppat.1006811.s002.docx]

**S1 Table. Steady-state kinetic analysis of AldA, AldB, and AldC**

| Protein | Substrate | *k*_cat_ (min^-1^) | *K*_m_ (μM) | *k*_cat_/*K*_m_ (M^-1^ s^-1^) |
| --- | --- | --- | --- | --- |
| AldA | IAAld | 234 ± 21 | 119 ± 37 | 32,770 |
|  | NAD^+^ | 194 ± 9 | 42 ± 8 | 77,400 |
|  | NADP^+^ | 30 ± 1 | 1,960 ± 182 | 252 |
| AldB | IAAld | 8.7 ± 0.2 | 595 ± 39 | 244 |
|  | NAD^+^ | 2.1 ± 0.1 | 33.4 ± 9.2 | 1,044 |
| AldC | IAAld | 3.6 ± 0.2 | 1,280 ± 161 | 46 |
|  | NAD^+^ | 0.40 ± 0.01 | 445 ± 99 | 14 |

Assays were performed as described in the experimental methods. All values are expressed as a mean ± SEM (*n* = 3).
