# Supplementary material for: Indole-3-acetaldehyde dehydrogenase-dependent auxin synthesis contributes to virulence of Pseudomonas syringae strain DC3000
Source: PLoS Pathog. 2018 Jan 2;14(1):e1006811. doi: 10.1371/journal.ppat.1006811 (PMC5766252; doi:10.1371/journal.ppat.1006811)
Supplement: S2 Table — (DOCX) [file ppat.1006811.s003.docx]

**S2 Table. Summary of crystallographic statistics for AldA**

| Crystal | AldA | AldA•NAD^+^ | AldA•NAD^+^•IAA |
| --- | --- | --- | --- |
| Space group | P22_1_2_1_ | P22_1_2_1_ | P22_1_2_1_ |
| Cell dimensions | *a*=80.89 Å, *b*=109.1 Å, *c*= 143.9 Å | *a*=80.68 Å, *b*=84.75 Å, *c*=166.8 Å | *a*=80.80 Å, *b*=84.88 Å, *c*=166.9 Å |
| Data Collection |  |  |  |
| Wavelength (Å) | 0.979 | 0.979 | 0.979 |
| Resolution range (Å)  (highest shell) | 40.5-2.09  (2.17-2.09) | 37.8-1.93  (2.00-1.93) | 45.83-2.09  (2.17-2.03) |
| Reflections  (total/unique) | 557,792 / 75,921 | 440,796 / 85,788 | 392,180 / 68,374 |
| Completeness  (highest shell) | 99.9% (99.3%) | 98.7% (97.2%) | 99.8% (98.4%) |
| <I/σ> (highest shell) | 34.9 (2.0) | 14.0 (2.5) | 12.8 (2.2) |
| R_sym_^a^ (highest shell) | 5.3% (67.1%) | 9.9% (72.4%) | 10.0% (62.2%) |
| Refinement |  |  |  |
| R_cryst_^b^ / R_free_^c^ | 18.2% / 21.3% | 14.6% / 18.1% | 15.1% / 18.9% |
| No. of protein  atoms | 6,425 | 7,386 | 7,386 |
| No. of waters | - | 88 | 114 |
| No. of ligand  atoms | 337 | 1,209 | 855 |
| R.m.s. deviation,  bond lengths (Å) | 0.008 | 0.005 | 0.007 |
| R.m.s. deviation,  bond angles (°) | 1.041 | 0.985 | 1.009 |
| Avg. B-factor (Å^2^):  protein, water,ligand | 51.0, -, 53.6 | 15.7, 25.0, 31.4 | 19.9, 36.8, 32.5 |
| Stereochemistry:  most favored,  allowed, outliers | 96.0, 3.4, 0.6 | 97.0, 2.8. 0.2 | 97.0, 2.9, 0.1 |

^a^R_sym_ = Σ|I_h_ - <I_h_>|/ΣI_h_, where <I_h_> is the average intensity over symmetry.

^b^R_cryst_ = Σ|F_o_ - <F_c_>|/ΣF_o_, where summation is over the data used for refinement.

^c^R_free_ is defined the same as R_cryst_, but was calculated using 5% of data excluded from refinement.
