# Supplementary material for: Indole-3-acetaldehyde dehydrogenase-dependent auxin synthesis contributes to virulence of Pseudomonas syringae strain DC3000
Source: PLoS Pathog. 2018 Jan 2;14(1):e1006811. doi: 10.1371/journal.ppat.1006811 (PMC5766252; doi:10.1371/journal.ppat.1006811)
Supplement: S3 Table — (DOCX) [file ppat.1006811.s004.docx]

**S3 Table: Bacterial strains and vectors used in this study**

**Strain or Plasmid Characteristics Reference or Source**

***Pseudomonas syringae* strains**

*P. syringae* pv. *tomato* DC3000 Derivative of NCPPB1106; Rif^r^ ([Cuppels, 1986](#_ENREF_14))

*aldA::*pJP5603 *PSPTO_0092* disrupted with pJP5603; Rif^r^, Km^r^ This study

*aldB::*pJP5603 *PSPTO_2673* disrupted with pJP5603; Rif^r^, Km^r^ This study

*aldC::*pJP5603 *PSPTO_3644* disrupted with pJP5603; Rif^r^, Km^r^ This study

*aldB::*pJP5603Tet *PSPTO_2673* disrupted with pJP5603-Tet; Rif^r^, Tet^r^ This study

*aldA*::pJP5603 *aldB*::pJP5603Tet double mutant; Rif^r^, Km^r^, Tet^r^ This study

*aldA*::pJP5603 p*AldA* *aldA* mutant with complementing clone; Rif^r^, Km^r^, Tet^r^ This study

***Escherichia coli* strains**

DH5α recA, lacZΔM15 Invitrogen

BL21(DE3) F^-^ *ompT* *hsdS_B_(r_B_^-^ m_B_^-^)* *gal* *dcm* (DE3) Novagen

DH5α λpir recA, lacZΔM15, λpir ([Miller and Mekalanos, 1988](#_ENREF_33))

MM294A (pRK2013) Triparental mating helper strain; Cm^r^ ([Finan et al., 1986](#_ENREF_20))

***Plasmids***

pCR BLUNT-II-TOPO Cloning Vector; Km^r^ Invitrogen

pET-21a Expression vector; Amp^r^

pET-28a Expression vector; Km^r^ Novagen

pTOPO-0092 *PSPTO_0092* CDS in pTOPO; Km^r^ This study

pET-21a-0092 *PSPTO_0092* CDS in pET21a; Amp^r^ This study

pET-28a-0092 *PSPTO_0092* CDS in pET28a; Km^r^ This study

pTOPO-0728 *PSPTO_0728* CDS in pTOPO; Km^r^ This study

pET-21a-0728 *PSPTO_0728* CDS in pET21a; Amp^r^ This study

pTOPO-2673 *PSPTO_2673* CDS in pTOPO; Km^r^ This study

pET-21a-2673 *PSPTO_2673* CDS in pET21a; Amp^r^ This study

pET-28a-2673 *PSPTO_2673* CDS in pET28a; Km^r^ This study

pTOPO-3064 *PSPTO_3064* CDS in pTOPO; Km^r^ This study

pET-21a-3064 *PSPTO_3064* CDS in pET21a; Amp^r^ This study

pTOPO-3323 *PSPTO_3323* CDS in pTOPO; Km^r^ This study

pET-21a-3323 *PSPTO_3323* CDS in pET21a; Amp^r^ This study

pTOPO-3364 *PSPTO_3364* CDS in pTOPO; Km^r^ This study

pET-21a-3364 *PSPTO_3364* CDS in pET21a; Amp^r^ This study

pET-28a-3644 *PSPTO_3644* CDS in pET28a; Km^r^ This study

pJP5603 Suicide vector; Km^r^ ([Penfold and Pemberton, 1992](#_ENREF_42))

pJP5603Tet pJP5603 with Tet^r^ in place of Km^r^; Tet^r^  This study

pTOPO-0092int Construct used to make *aldA*::pJP5603 strain; Km^r^ This study

pJP5603-0092int Construct used to make *aldA*::pJP5603 strain; Km^r^ This study

pTOPO-2673int Construct used to make *aldB*::pJP5603 strain; Km^r^ This study

pJP5603-2673int Construct used to make *aldB*::pJP5603 strain; Km^r^ This study pJP5603Tet-2673int Construct used to make *aldB*::pJP5603Tet strain; Km^r^ This study

pTOPO-3644int Construct used to make *aldC*::pJP5603 strain; Km^r^ This study

pJP5603-3644int Construct used to make *aldC*::pJP5603 strain; Km^r^ This study

pTOPO-0092comp Construct used to make p*AldA* complement clone; Km^r^ This study

pME6031 Replicating vector, stable in *P. syringae*; Tet^r^ ([Heeb et al., 2000](#_ENREF_23))

p*AldA* *PSPTO_0092* CDS and promoter region in pME6031; Tet^r^ This study
