# Supplementary material for: Indole-3-acetaldehyde dehydrogenase-dependent auxin synthesis contributes to virulence of Pseudomonas syringae strain DC3000
Source: PLoS Pathog. 2018 Jan 2;14(1):e1006811. doi: 10.1371/journal.ppat.1006811 (PMC5766252; doi:10.1371/journal.ppat.1006811)
Supplement: S4 Table — (DOCX) [file ppat.1006811.s005.docx]

**S4 Table. Related to Experimental Procedures. List of primers used in this study:**

| **For cloning** | | | |
| --- | --- | --- | --- |
| **Name** | **Sequence** | | **comments** |
| 0092NdeIF | 5’ ATATCATATGACCACCCTGACCC 3’ | | NdeI site underlined |
| 0092XhoIR | 5’ TTAACTCGAGTTACAACTTGATCCAGGTGG 3’ | | XhoI site underlined |
| 0728NdeIF | 5’ ATATCATATGCGTTACGCTCATCCCG 3’ | | NdeI site underlined |
| 0728XhoIR | 5’ TTAACTCGAGCTAGAAAAAACCCAGCGGG 3’ | | XhoI site underlined |
| 2673NdeIF | 5’ ATATCATATGAGCCTTACCCGCTTTCA3’ | | NdeI site underlined |
| 2673XhoIR | 5’ TTAACTCGAGTTAACGCATGACAAATGGATCAGG 3’ | | XhoI site underlined |
| 3064NdeIF | 5’ ATATCATATGCATGATCTTCTTACCGCAG 3’ | | NdeI site underlined |
| 3064XhoIR | 5’ TTAACTCGAGTCATTTTACGGTCTCGTCAACC 3’ | | XhoI site underlined |
| 3323NdeIF | 5’ ATATCATATGCCTAACATTCTCGGCC 3’ | | NdeI site underlined |
| 3323Xho1R | 5’ TTAACTCGAGTCACGCGACAGGCTGATC 3’ | | XhoI site underlined |
| 3644NdeIF | 5’ ATATCATATGACCACACAGCCCTTGAAC 3’ | | NdeI site underlined |
| 3644XhoIR | 5’ TTAACTCGAGTTACATCAAGTCAGCCGGAGC 3’ | | XhoI site underlined |
| 0092SacIF | 5’ ATATGAGCTCAAGGCCACCATGATCCGTTTC 3’ | | SacI site underlined |
| 0092XbaIR | 5’ TTAATCTAGAGCTTCATGTTCGATTCGCCC 3’ | | XbaI site underlined |
| 2673SacIF | 5’ ATATGAGCTCTCTGATCGCCGAGAACAAGG 3’ | | SacI site underlined |
| 2673XbaIR | 5’ TTAATCTAGACAGTTCCAGCGACAGTTTGG 3’ | | XbaI site underlined |
| 3644SacIF | 5’ ATATGAGCTCCCTGGGGTGTTCAATCTGGTC 3’ | | SacI site underlined |
| 3644XbaIR | 5’ TTAATCTAGACAAAAATCTCCTCACGGGCGATG3’ | | XbaI site underlined |
| 0092Xho1F | 5’ CTCGAGGCTGGATCCTTGCGCTGAATAC 3’ | | XhoI site underlined |
| 0092EcoRIR | 5’ GAATTCGGACCTGCGCAATTCTTTGCG 3’ | | EcoRI site underlined |
| **For genotyping** | | | |
| Name | Sequence | Organism and gene | |
| M13F | 5’ GTAAAACGACGGCCAG 3’ |  | |
| M13R | 5’ CAGGAAACAGCTATGACC 3’ |  | |
| 0092seqF | 5’ CGTACTGGTTGACCCACAC 3’ | *P. syringae* strain DC3000 PSPTO_0092 | |
| 0092seqR | 5’ GAACAACGCGCCCAAAATC 3’ | *P. syringae* strain DC3000 PSPTO_0092 | |
| 2673seqF | 5’ ATCCCTGAACGAATGTCCCG 3’ | *P. syringae* strain DC3000 PSPTO_2673 | |
| 2673seqR | 5’ TGAGCCTTACCCGCTTTCAG 3’ | *P. syringae* strain DC3000 PSPTO_2673 | |
| 3644seqF | 5’ CAAACCCCACAATCGCCTTG 3’ | *P. syringae* strain DC3000 PSPTO_3644 | |
| 3644seqR | 5’ TGGACCTGGCCGCTCTATC 3’ | *P. syringae* strain DC3000 PSPTO_3644 | |
|  |  |  | |
| **For quantitative real-time PCR qRT-PCR** | | | |
| Name | Sequence | Organism and gene | |
| PP2A3F | 5’ AACGTGGCCAAAATGATGC 3’ | *A. thaliana*  AT1G13320 | |
| PP2A3R | 5’ AACCGCTTGGTCGACTATCG 3’ | *A. thaliana* AT1G13320 | |
| ICS1F | 5’ TAGAGGAATGTATGCGGGAC 3’ | *A. thaliana* AT1G74710 | |
| ICS1R | 5’ TCTGAAGATGGGTCACTTCC 3’ | *A. thaliana* AT1G74710 | |
| PR1F | 5’ GGAGCTACGCAGAACAACTAA 3’ | *A. thaliana* AT2G14610 | |
| PR1R | 5’ CCCACGAGGATCATAGTTGCAACTGA 3’ | *A. thaliana* AT2G14610 | |
