# Supplementary figures and images for: Indole-3-acetaldehyde dehydrogenase-dependent auxin synthesis contributes to virulence of Pseudomonas syringae strain DC3000
Source: PLoS Pathog. 2018 Jan 2;14(1):e1006811. doi: 10.1371/journal.ppat.1006811 (PMC5766252; doi:10.1371/journal.ppat.1006811)

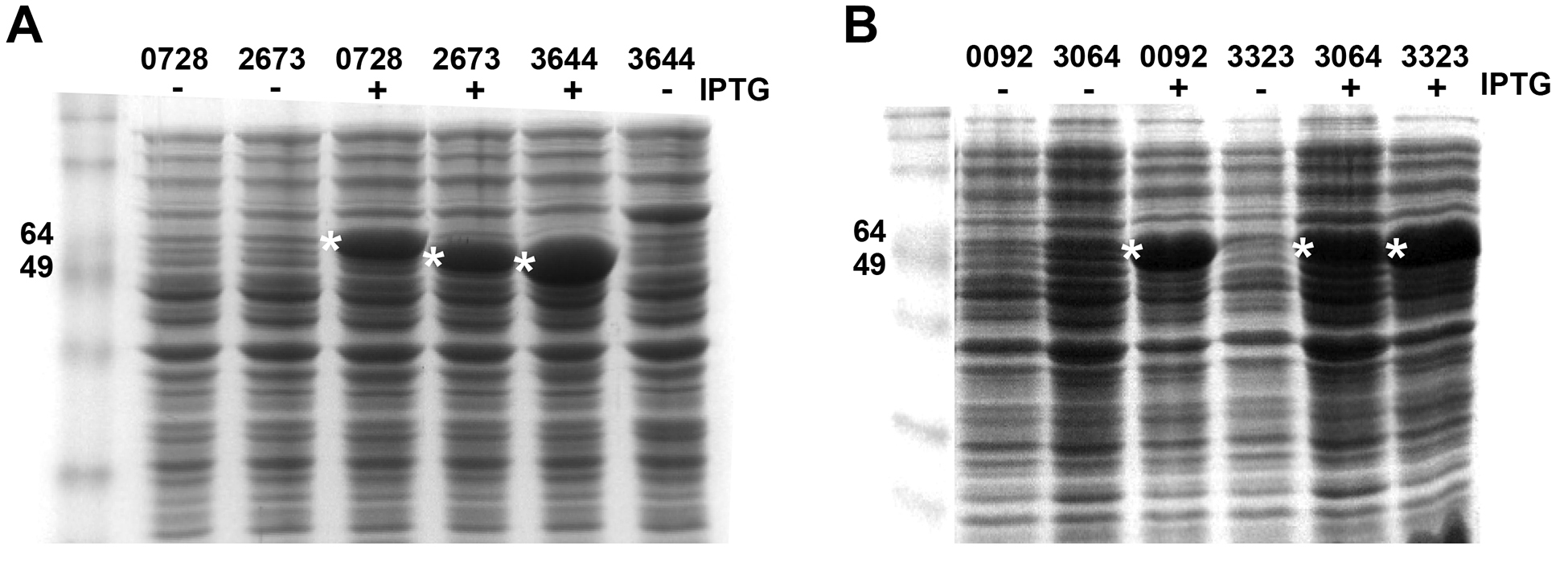

Supplement: S1 Fig — Putative DC3000 Ald proteins were expressed from the pET-21a vector in E. coli. Protein expression was induced with IPTG (1 mM final concentration). Protein lysates (20 μl) were separated by electrophoresis on a 12% polyacrylamide gel and visualized by staining with Coomassie blue. The Ald proteins (expected size ~53kDa) are indicated by asterisks. (TIF) [file ppat.1006811.s006.tif]

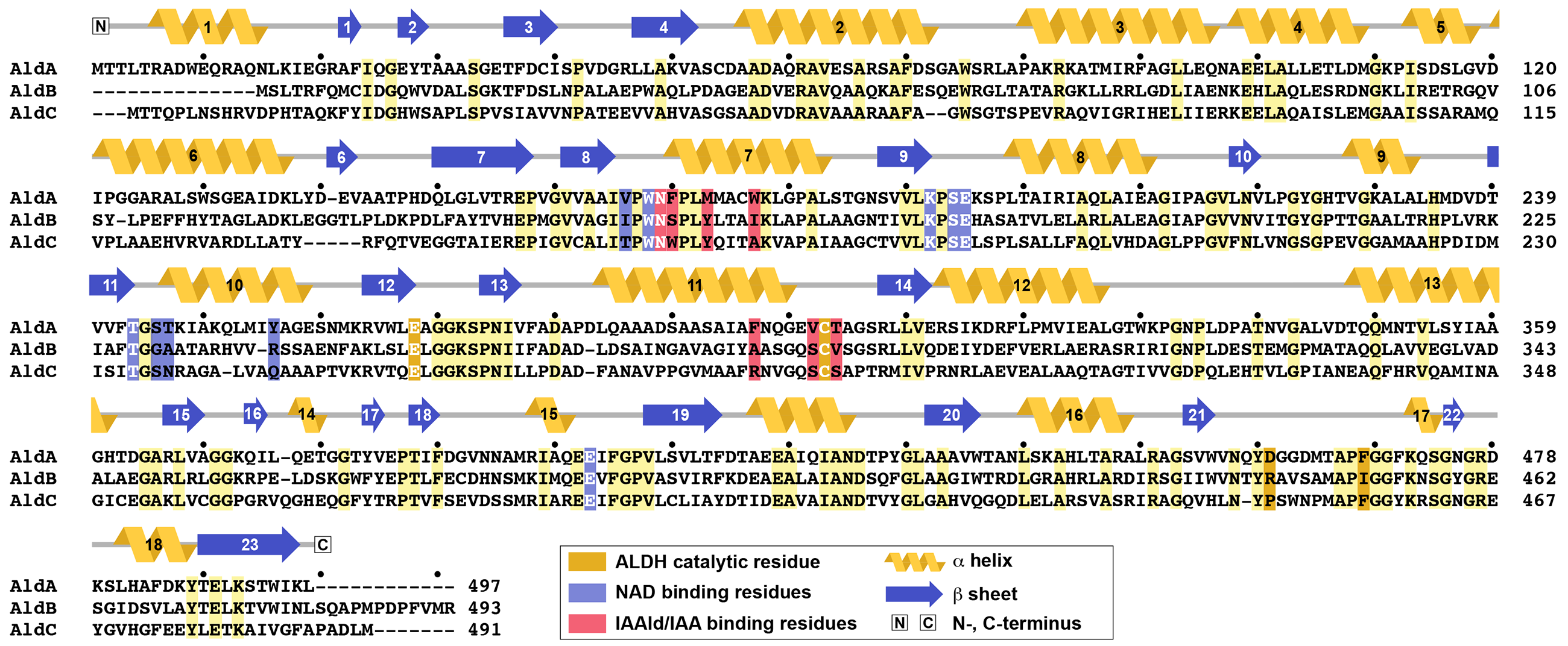

Supplement: S2 Fig — Alignment was performed using the Multalign server (multalin.toulouse.inra.fr). Residues required for catalysis (gold), NAD(H) binding (blue), and IAAld/IAA binding (red) are indicated by colored boxes. Invariant residues are highlighted by yellow boxes. The secondary structure of AldA is shown above the sequence alignment. (TIF) [file ppat.1006811.s007.tif]

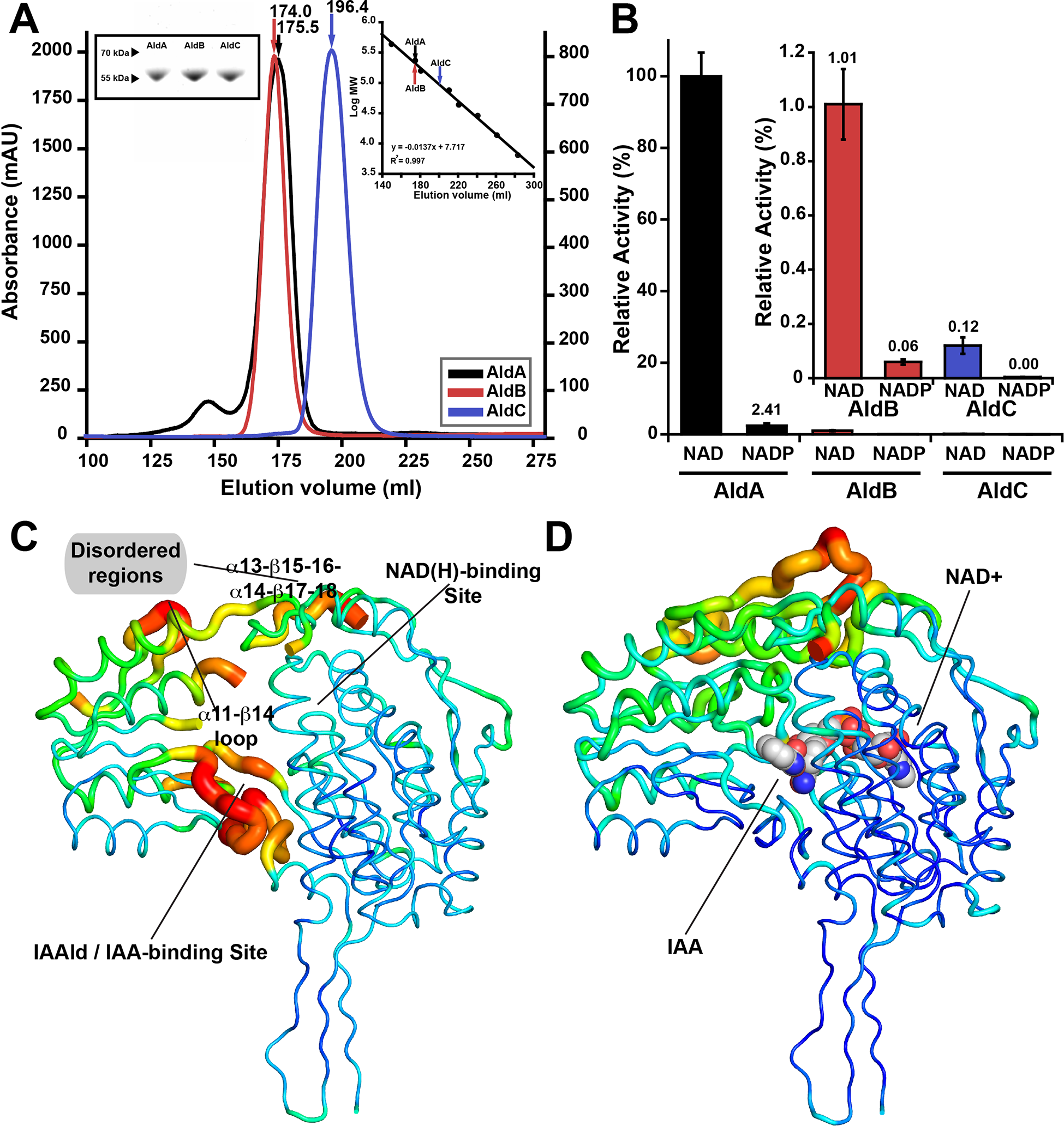

Supplement: S3 Fig — A) Size-exclusion chromatography of AldA (black), AldB (red), and AldC (blue). Each protein was analyzed on a Superdex-200 26/60 FPLC column. Elution volumes are indicated. The inset on the left shows SDS-PAGE of purified AldA-C proteins. Positions of molecular weight markers are indicated. The inset on the right shows the molecular weight calibration of the size-exclusion column. The following standards were used to calibrate the column: ferritin (440 kDa), catalase (232 kDa), aldolase (158 kDa), conalbumin (75 kDa), ovalbumin (44 kDa), carbonic anhydrase (29 kDa), ribonuclease (13.7 kDa), and aprotinin (6.5 kDa). B) Specific activities of AldA, AldB and AldC were determined using standard assay conditions using IAAld and either NAD+ or NADP+ as substrates, as described in the experimental methods. Relative activity is shown using AldA with IAAld and NAD+ as 100% (3.52 μmol min-1 mg protein-1). The inset zooms in on the lower specific activities for AldB and AldC. Values are expressed as a mean ± SEM (n = 3). C & D) Relative B-factors for the AldA apoenzyme (C) and AldA•NAD+•IAA complex (D) are shown. Low relative B-factors are indicated by thin blue cartoon and highest B-factors by the thicker red cartoon. Positions of the cofactor and substrate binding sites in the monomer are indicated. Disordered regions in the apoenzyme structure are also indicated. (TIF) [file ppat.1006811.s008.tif]

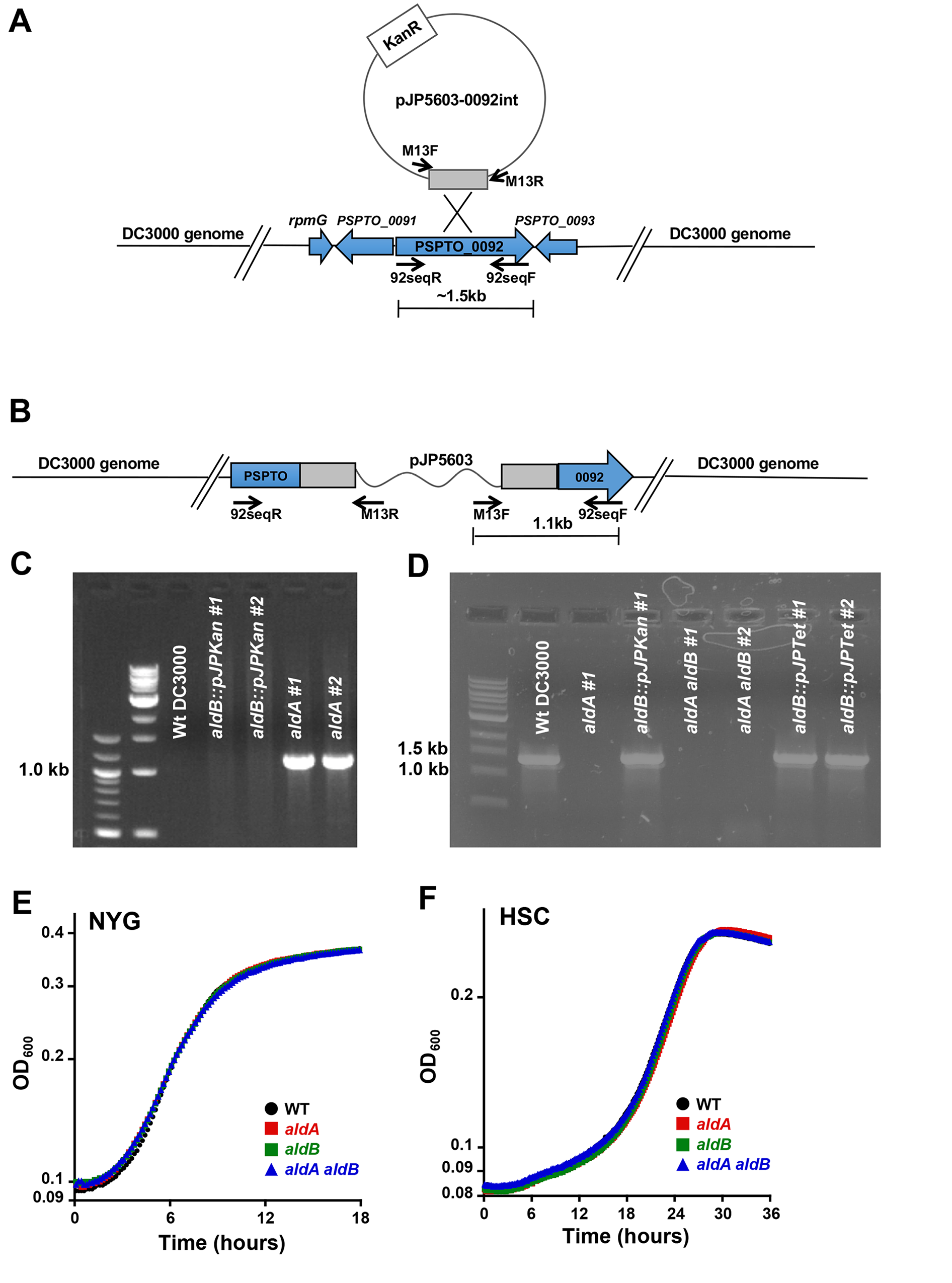

Supplement: S4 Fig — A) The PSPTO_0092 region of DC3000 genome showing neighboring genes (blue arrows) and plasmid pJP5603-0092int containing a ~530 bp internal fragment of PSPTO_0092 (gray box) used to generate the aldA mutant. B) Schematic diagram illustrating the result of a single homologous recombination event between pJP5603-0092int and the chromosomal copy of PSPTO_0092, leading to disruption of the gene. C) Amplification of the plasmid-disrupted PSPTO_0092 gene using primer pairs M13F and 0092seqF. D) Amplification of wild-type PSPTO_0092 using primer pairs 0092 seq F/R. The genotypes of the strains are indicated. aldB:pJPKan and aldB:pJPTet refers to mutants generated by integration of pJP5603-2673int and pJP5603Tet-2673int, respectively. Primer pairs used in PCR reactions shown in panels C and D are illustrated by arrow heads in panels A & B (S4 Table). E) Growth of WT DC3000 (black), aldA (red), aldB (green), and the aldA aldB double mutant (blue) at 30°C in NYG media. Values are an average of three biological replicates. Exponentially growing cells were diluted to OD600nm = 0.025 to start the cultures at a uniform cell density. Cell growth (OD600nm) was monitored every 10 minutes for 18 hours using an EPOCH2 microplate reader (BioTek). The aldA, aldB:pJPTet, and aldA aldB double mutants exhibited similar growth compared to the WT DC3000 at all time points. Similar results were obtained in a second independent experiment. F) Growth of WT DC3000 (black), aldA (red), aldB:pJPTet (green), and the aldA aldB double mutant (blue) at 30°C in HSC media. Values are an average of three biological replicates. Exponentially growing cells were diluted to OD600nm = 0.050 to start the cultures at a uniform cell density. Cell growth (OD600nm) was monitored at an interval of 10 minutes for 36 hours using EPOCH2 microplate reader (BioTek). The aldA, aldB, and the aldA aldB mutants exhibited similar growth compared to the WT DC3000 at all time points. Similar results were obtained in a second i [file ppat.1006811.s009.tif]

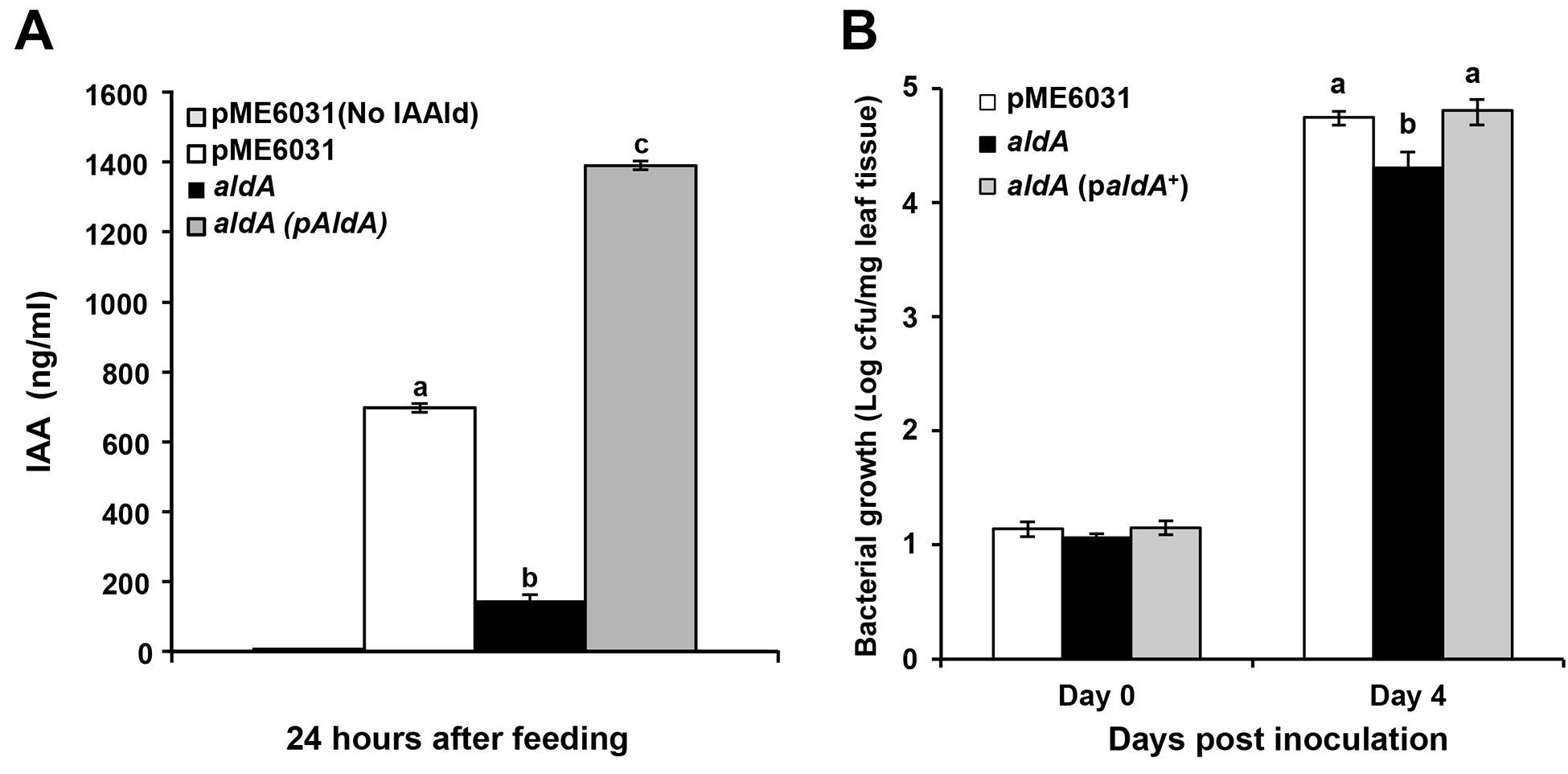

Supplement: S5 Fig — A) Measurement of IAA accumulation in supernatants of DC3000 (pME6031, empty vector control), aldA carrying pME6031, and aldA carrying the corresponding complementing plasmid, pAldA, grown for 48 hrs in HSC media supplemented with 0.25 mM IAAld. IAA values are an average of three biological replicates ± SEM. B) Growth of DC3000 (pME6031) aldA carrying pME6031, and aldA carrying the corresponding complementing plasmid pAldA in A. thaliana plants after syringe infiltration (OD600nm = 1x10^-4^). Values are an average of three biological replicates ± SEM. Letters indicate significant difference between samples within a given time point (p<0.05). (TIF) [file ppat.1006811.s010.tif]
